# Metagenomic next-generation sequencing confirmed pseudorabies virus encephalitis complicated with central visual impairment: a case report

**DOI:** 10.3389/fneur.2026.1954544

**Published:** 2026-10-15

**Authors:** Yonghu Zhang, Xiaojing Zhang, Xue Wei, Wenwen Yu, Ning Ma, Weihai Liu, Yafeng Zheng, Hongyu Jia

**Affiliations:** 1 Department of Infectious Diseases, Beilun People's Hospital, Ningbo, Zhejiang, China; 2 Willingmed Technology (Beijing) Co. Ltd., Beijing, China

**Keywords:** blindness, metagenomic next-generation sequencing, pseudorabies virus, viral encephalitis, zoonosis

## Abstract

Pseudorabies virus (PRV) is an alphaherpesvirus for which domestic swine serve as the sole natural reservoir. Human PRV infection has historically been regarded as extremely rare. Since 2017, however, an increasing number of severe human PRV encephalitis cases have been identified in China, pre-dominantly among individuals with occupational exposure to pigs or pork products. The condition has been associated with substantial morbidity and mortality and remains under recognized in clinical practice. We report a 53-year-old male dockworker who presented with a 9-day history of fever followed by progressive severe headache. PRV infection was confirmed by metagenomic next-generation sequencing (mNGS) of cerebrospinal fluid (CSF). Brain magnetic resonance imaging revealed signal abnormalities involving the right insular cortex, temporal lobe, hippocampus, and amygdala, consistent with encephalitis. The patient received intravenous acyclovir (500 mg every 8 h for 23 days), corticosteroids, and intravenous immunoglobulin. Although his level of consciousness gradually improved, he developed permanent bilateral blindness with no light perception. At 1-year follow-up, he remained alive but with persistent blindness; neuroimaging demonstrated encephalomalacia in the right temporal and insular regions. PRV can cause devastating human encephalitis with visual impairment as a frequent and serious complication, reported in up to 60% of published cases. Our case is notable in that bilateral blindness emerged after recovery of consciousness, without evidence of peripheral ocular pathology, suggesting a pre-dominantly central visual pathway injury. CSF mNGS has become a key tool for timely diagnosis of rare viral encephalitides. Clinicians should consider PRV encephalitis in patients with unexplained encephalitis and a history of swine exposure, and pursue early mNGS testing to facilitate prompt aetiological diagnosis and antiviral therapy.

## 1 Introduction

Pseudorabies virus (PRV; family Herpesviridae, subfamily Alphaherpesvirinae, genus Varicellovirus), also called Suid Herpesvirus 1 (SuHV-1), or Aujeszky's Disease Virus, is an enveloped, neurotropic double-stranded DNA virus that causes Aujeszky's disease in domestic pigs. Swine are the sole natural reservoir and the only species in which the virus can establish productive, persistent infection (1, 2). In other mammals (including cattle, sheep, dogs, and cats), PRV infection is almost uniformly fatal, typically manifesting with severe neurological signs and intense pruritus, a condition historically referred to as “mad itch” (1). Although PRV is not a classical zoonotic pathogen capable of efficient human-to-human transmission, sporadic human infections have been documented following occupational exposure to infected pigs or contaminated materials (1–3).

Historically, human PRV infection was considered exceedingly rare. Early case reports were limited to localized ocular or cutaneous infections in veterinarians and slaughterhouse workers, and no cases of encephalitis appeared in the literature until the mid-2010s. In 2017, the application of metagenomic next-generation sequencing (mNGS) led to the first reported cluster of human PRV encephalitis cases in China. Multiple subsequent publications have revealed an emerging pattern of severe PRV encephalitis in pig-farming regions. A recent comprehensive review in The Lancet Microbe (2026) documented 31 laboratory-confirmed human PRV cases in China between 2017 and 2023, raising public health concerns about the enhanced human pathogenicity of circulating PRV strains (1). Around 2011, a novel highly virulent PRV variant emerged that carries specific insertional mutations in key virulence genes (gE and gC). These mutations have been shown to overcome the protection conferred by the Bartha-K61 vaccine for swine and are thought to underpin the variant's increased neurotropism and capacity for cross-species transmission (2, 4).

Clinically, human PRV encephalitis closely resembles herpes simplex virus type 1 (HSV-1) encephalitis, with fever, altered consciousness, seizures, and limbic system involvement on neuroimaging (3, 5). This phenotypic overlap, compounded by the absence of PRV-specific testing in routine diagnostic algorithms, frequently leads to misdiagnosis or delayed diagnosis. mNGS has become a key diagnostic tool for confirming PRV etiology (6, 7).

Herein, we report a case of severe PRV encephalitis confirmed by CSF mNGS, in which the patient developed permanent bilateral blindness after recovery of consciousness without evidence of peripheral ocular pathology. This presentation suggests a pre-dominantly central visual pathway injury. The case extends current understanding of PRV-associated visual complications and underscores the importance of early mNGS-based diagnosis in suspected cases.

## 2 Case presentation

A 53-year-old male dockworker was admitted to the Department of Neurology at Beilun People's Hospital, Ningbo, on 20 January 2024, with a chief complaint of fever for 9 days and recurrent worsening headache for 3 days. His medical history was unremarkable except for an appendectomy 20 years previously; he had no history of hypertension, diabetes, or neurological disorders. He reported a 3-year history of alcohol consumption. The clinical course of the patient is shown in Figure 1.

**Figure 1:**
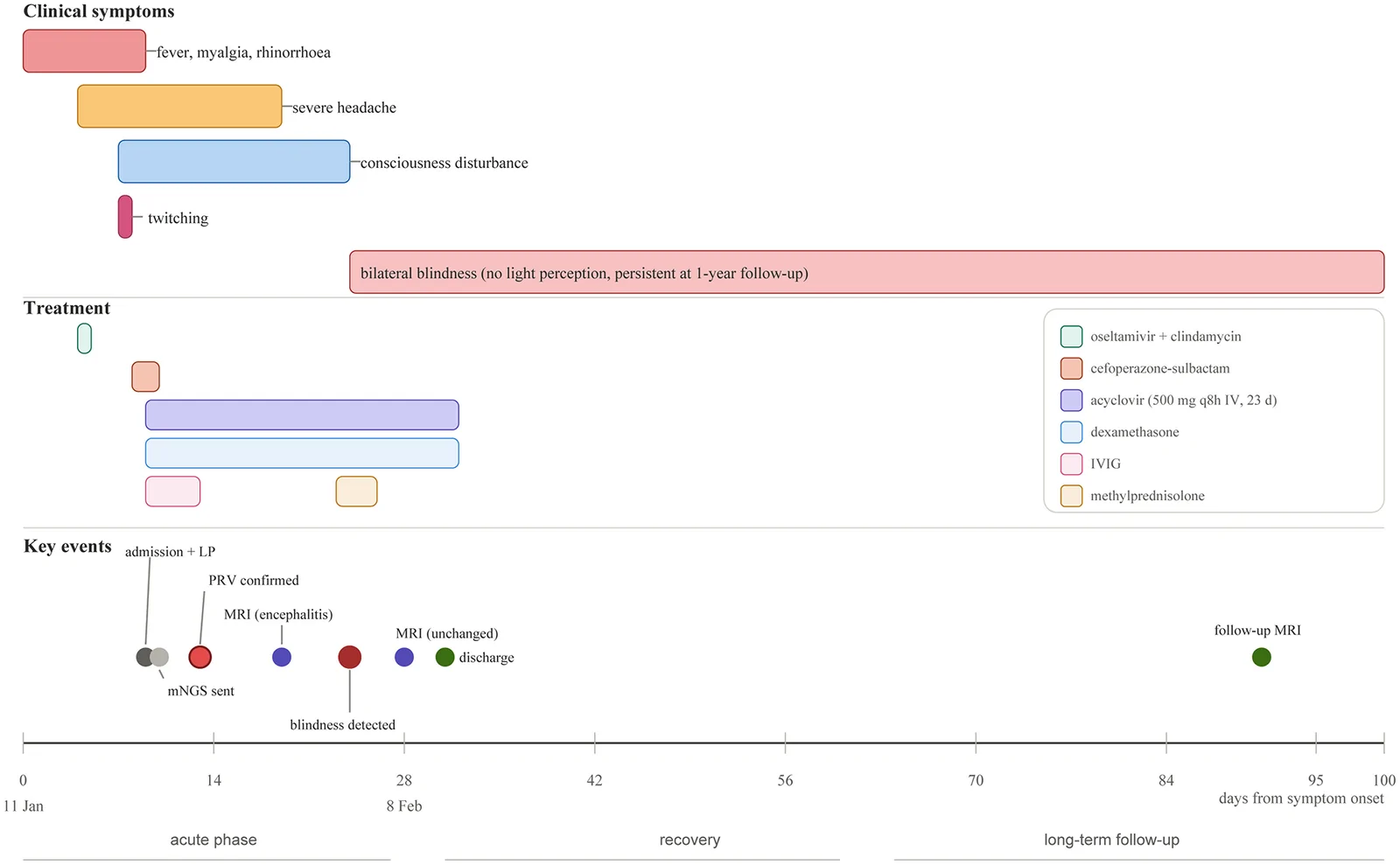
Clinical timeline of the patient's disease course. The timeline illustrates key clinical events from symptom onset (11 January 2024) through admission, diagnosis, treatment, and 1-year follow-up. PRV, pseudorabies virus; mNGS, metagenomic next-generation sequencing; MRI, magnetic resonance imaging; IVIG, intravenous immunoglobulin.

On 11 January 2024 (9 days before admission), the patient developed fever (maximum 39.0 °C) accompanied by generalized myalgia, rhinorrhoea, chills, fatigue, and anorexia. His symptoms partially improved after 4 days of treatment at a local clinic. On 17 January 2024 (3 days before admission), fever recurred (maximum 38.6 °C), together with severe headache characterized by a pressing sensation over the bilateral temporal and frontal regions; the headache mildly subsided with defervescence, and intermittent nausea occurred. On 18 January 2024, he visited the Second People's Hospital of Beilun, where laboratory tests showed a white blood cell count (WBC) of 9.73 × 10^9^/L (Ref. 3.5–9.5 × 10^9^/L), neutrophils (NEUT) 6.98 × 10^9^/L (Ref: 1.80–6.30 × 10^9^/L), and high-sensitivity C-reactive protein (hsCRP) 1 mg/L (Ref: 0–10 mg/L). Chest computed tomography (CT) revealed a punctate calcification in the apex of the right lung. Treatment with oseltamivir and clindamycin was initiated, but the headache continued to worsen. On 20 January 2024 (day of admission), a repeat blood test at the same hospital showed marked leukocytosis (WBC, 24.06 × 10^9^/L), and the patient was transferred to our emergency department. Suspecting intracranial infection, we admitted him to the neurology ward for further evaluation.

On admission, vital signs were: temperature 38.3 °C, pulse 110 beats per minute, respiratory rate 22 breaths per minute, and blood pressure 170/99 mmHg. The patient was conscious but lethargic, with slow responses and poor cooperation. Mild scleral icterus was noted. Pupils were equal, round, and reactive to light. Extraocular movements were grossly intact, without nystagmus or diplopia. Facial sensation was symmetrical, and no facial asymmetry or deviation of the mouth was observed. The tongue was midline, and the gag reflex was preserved, without choking or dysphagia. Neck resistance could not be reliably assessed due to poor cooperation. Motor examination revealed grade 5/5 strength in all four limbs, with normal muscle tone. A right Babinski sign was equivocal; Kernig's sign and finger-to-nose testing could not be evaluated because of poor cooperation. Laboratory findings: WBC 24.06 × 10^9^/L (Ref. 3.5–9.5 × 10^9^/L), NEUT 22.12 × 10^9^/L (Ref: 1.80–6.30 × 10^9^/L), lymphocytes 0.73 × 10^9^/L (Ref: 1.10–3.20 × 10^9^/L); hemoglobin 163 g/L (Ref: 120–175 g/L); platelets 342 × 10^9^/L (Ref: 125–300 10^9^/L); hsCRP 1 mg/L (Ref: 0–10 mg/L). Initial lumbar puncture yielded cerebrospinal fluid (CSF) with a white blood cell count of 21/μl and a mononuclear cell proportion of 16.0%; CSF glucose 5.21 mmol/L; CSF chloride 105.4 mmol/L. Non-contrast head CT showed no obvious parenchymal abnormalities, though bilateral ethmoid sinusitis was noted. Shortly after lumbar puncture, the patient's condition fluctuated with restlessness, generalized twitching, and blurred consciousness. He was treated with mannitol for intracranial pressure reduction, corticosteroids (Dexamethasone injection 10 mg daily, Jan 20–24), fluid replacement, and antipyretics, after which his condition gradually stabilized.

On 21 January 2024, the patient's condition deteriorated. He became confused, with fixed gaze and unresponsiveness to verbal stimuli; pupils remained reactive to light, and withdrawal to painful stimuli was preserved. Temperature was 38.2 °C. A repeat lumbar puncture was performed, and CSF was sent for mNGS. Empirical antibiotic therapy with cefoperazone-sulbactam (2 g every 8 h, intravenous) was initiated. On 22 January, the patient remained critically ill with persistent high fever and somnolence; T-SPOT.TB was negative, and acid-fast bacilli were not detected in CSF. On 23 January, he was somnolent with high fever and could not cooperate with examination. On 24 January, D-dimer was elevated and coagulation parameters were otherwise abnormal. He was transferred to the First Affiliated Hospital of Zhejiang University for further management.

At the tertiary center, repeat CSF analysis showed clear, colorless fluid; Pandy's test was negative; red blood cells 20/μl; nucleated cells 4/μl. CSF biochemistry: adenosine deaminase 2 U/L, chloride 111 mmol/L, glucose 3.7 mmol/L, lactate dehydrogenase 32 U/L, protein 0.51 g/L. CSF mNGS returned positive for Suid herpesvirus 1 (PRV). Antiviral therapy with acyclovir 500 mg every 8 h intravenously was commenced on 24 January 2024 and continued until 15 February 2024. Dexamethasone was administered for inflammation control (10 mg twice daily from 24 January, reduced to 10 mg once daily from 29 January until 15 February), and intravenous immunoglobulin (15 g once daily) was given from 24 to 30 January 2024.

On 2 February 2024, brain MRI (plain + contrast + diffusion-weighted imaging) showed swelling and signal changes in the right insular cortex, right temporal lobe, right hippocampus, and amygdala, consistent with encephalitis; mild bilateral subcortical white matter hyperintensities (Fazekas grade 1) were also noted (Figure 2A). From 5 February onward, the patient became fully conscious, could answer questions appropriately, and mobilized independently. However, he reported bilateral blindness with no light perception, and pupillary light reflexes were absent. Central nervous system demyelination was suspected, and pulsed methylprednisolone (500 mg once daily intravenously, 6–10 February 2024) was administered without visual improvement. Follow-up MRI on 14 February 2024 (Figure 2B) showed signal abnormalities in the bilateral insulae, right temporal lobe, right hippocampus, and amygdala, largely unchanged from the previous scan; white matter hyperintensities remained stable.

**Figure 2:**
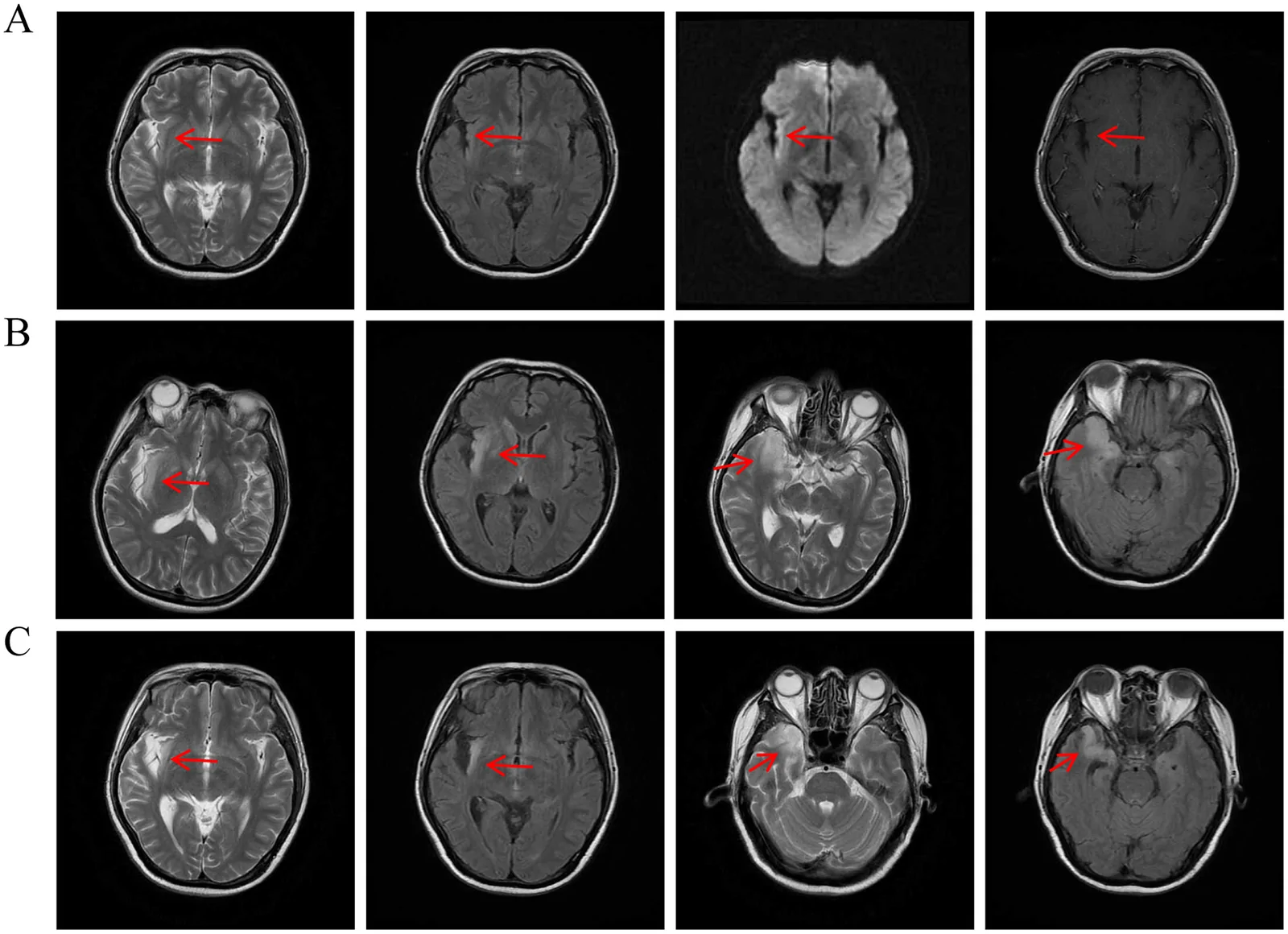
Serial brain MRI findings. **(A)** Brain MRI obtained on 21 January 2024 (10 days after symptom onset). A strip-like abnormal signal is visible in the superficial right insular cortex (arrow), appearing slightly hyperintense on T2WI and FLAIR, hyperintense on DWI, and without obvious enhancement on post-contrast imaging. **(B)** Brain MRI obtained on 4 March 2024. The right insular lesion has expanded and extends to involve the right temporal lobe. **(C)** Brain MRI obtained on 9 May 2024. The extent of the right insular and right temporal lobe lesions has decreased compared with the 4 March examination. T2WI, T2-weighted imaging; FLAIR, fluid-attenuated inversion recovery; DWI, diffusion-weighted imaging.

The patient was discharged home and continued acyclovir and tapering methylprednisolone under local supervision. At 1-year follow-up (May 2024), he remained alive but with persistent bilateral blindness (no light perception); he reported mild pain on raising his upper arms but had no other significant complaints. Repeat contrast-enhanced brain MRI on 9 May 2024 showed residual abnormal signals in the right superficial insula and right temporal lobe (Figure 2C), suggestive of encephalitic changes, which had improved compared with the MRI of 4 March 2024; periventricular white matter hyperintensities were unchanged.

## 3 Discussion

We report a case of severe PRV encephalitis confirmed by CSF mNGS in a 53-year-old male dockworker. The patient survived the acute phase but was left with permanent bilateral blindness, a debilitating sequela that persisted through 1-year follow-up. This case adds to the growing literature on emerging human PRV infections in China and highlights several clinically relevant points regarding epidemiology, diagnosis, visual complications, and therapeutic challenges.

PRV has long been enzootic in Chinese pig herds. The emergence, around 2011, of a highly virulent variant carrying insertional mutations in the gE and gC genes has coincided with human spillover events. This variant not only escapes protection conferred by the Bartha-K61 vaccine, a live-attenuated vaccine widely used for immunization of swine, but also exhibits enhanced neurotropism, a feature considered the molecular basis for its recently recognized capacity to infect humans (2, 4). It is important to note that currently available PRV vaccines are intended exclusively for veterinary use in pigs; no vaccine has been approved for human use against PRV infection to date. While vaccination remains a cornerstone of disease control in swine populations, it has an indirect role in reducing the viral reservoir and thus potentially lowering the risk of zoonotic spillover to humans (8).

Based on the keywords “pseudorabies virus encephalitis” or “suid herpesvirus encephalitis,” we searched the NCBI, Google Scholar, and Wanfang databases and included only cases in which the infection was confirmed by molecular testing, ultimately aggregating data from 27 patients (Table 1). The patients were pre-dominantly middle-aged males (male:female = 25:2), with ages ranging from young adulthood to 68 years. Occupationally, the cohort was highly concentrated in the swine industry chain, including pig farmers, butchers, veterinarians, pork vendors, and slaughterhouse workers, consistent with previously reported epidemiological profiles. Geographically, the cases originated from multiple major livestock-rearing provinces, including Shandong, Henan, Hebei, Hubei, Jiangxi, Guangdong, Beijing, Zhejiang, Anhui, Hunan, and Inner Mongolia, suggesting a sporadic distribution pattern that overlaps with intensive pig-farming regions across China. Our patient was a dockworker rather than a member of a traditional high-risk group such as farmers, veterinarians, or slaughterhouse workers, suggesting that the risk spectrum may be wider than previously appreciated. Although he had no habit of consuming raw pork, his daily work involved handling a variety of imported and exported goods; the exact route of exposure remained unidentified, representing a limitation of our report.

**Table 1:** The summarization of clinical features from reported patients with human PRV encephalitis.

Case	References	Age(year)	Sex	Occupation	Residence	Symptoms	Diagnostic method	Treatments	Outcomes
1	Current study	53	M	Dockworker	Zhejing	Fever, headache, myalgia, rhinorrhoea, chills, fatigue, and anorexia, disturbed consciousness, eye involvement (Bilateral blindness)	CSF NGS	Acyclovir, dexamethasone, IVIG	Bilateral blindness, mild pain on raising his upper arms
2	(32)	51	F	Housewife	Jiangxi	Fever, headache, seizures, disturbed consciousness, psychosis, respiratory failure	CSF NGS, Sanger sequencing	Acyclovir, linezolid, midazolam, sodium valproate	Coma, tracheostomy state, mRS:5
3	(32)	68	M	Swineherd	Jiangxi	Fever, seizures, disturbed consciousness, respiratory failure	CSF NGS, Sanger sequencing	Acyclovir, meropenem, midazolam, sodium valproate, IVIG	Died
4	(3)	mid-aged	M	Pig butcher	Shandong	Fever, seizures, apathy, dullness, cognitive decline, disturbed consciousness, respiratory failure, eye involvement (bilateral retinal necrosis)	CSF NGS, PCR	Acyclovir	Died
5	(3)	mid-aged	M	Swineherd	Hebei	Fever, seizures, cognitive decline, disturbed consciousness, respiratory failure, eye involvement (blindness)	CSF NGS, PCR	Acyclovir, foscarnet and methylprednisolone	Memory loss, hand tremor, mRS:3
6	(3)	young	M	Pig driver	Guangdong	Fever, seizures, disturbed consciousness, respiratory failure, renal fail	CSF NGS, PCR	Acyclovir and IVIG	Resumed consciousness and was able to obey commands, mRS:5
7	(3)	young	F	Pork dealer	Beijng	Fever, seizures, disturbed consciousness, respiratory failure, psychosis, eye involvement (bilateral fundus hemorrhage and papilledema)	CSF NGS, PCR	Acyclovir, foscarnet	Died
8	(2)	25	M	Veterinarian	Hubei	Fever, headache, seizures, cognitive impairment, disturbed consciousness, eye involvement (blindness)	CSF NGS, PRV envelope glycoprotein B antibody, glycoprotein E antibody, and neutralizing antibody	Ganciclovir, foscarnet	Permanent blindness, mRS:2
9	(2)	35	M	Pig butcher	Henan	Fever, headache, seizures, cognitive impairment, disturbed consciousness, respiratory failure	CSF NGS, PRV envelope glycoprotein B antibody, glycoprotein E antibody, and neutralizing antibody	Acyclovir, foscarnet	Mild memory impairment, mRS:2
10	(2)	49	M	Pig butcher	Henan	Fever, headache, seizures, cognitive impairment, disturbed consciousness, respiratory failure, eye involvement (retinal detachment)	CSF NGS, PRV envelope glycoprotein B antibody, glycoprotein E antibody, and neutralizing antibody	Ganciclovir, foscarnet	Minimally conscious, mRS:5
11	(2)	43	M	Veterinarian	Shandong	Fever, headache, disturbed consciousness, seizures, respiratory failure	CSF NGS, PRV envelope glycoprotein B antibody, glycoprotein E antibody, and neutralizing antibody	Acyclovir	Persistent vegetative status
12	(24)	55	M	Pig butcher	Neimenggu	Fever, headache, cough, seizures, disturbed con sciousness, respiratory failure, eye involvement (acute retinitis with bilateral lens opacity)	CSF NGS	Acyclovir, IVIG, and dexamethasone	Coma for 12 weeks, mRS:5
13	(24)	51	M	Pork cutter	Neimenggu	Fever, headache, apathy, seizures, disturbed con sciousness, psychosis, respiratory failure	CSF NGS	Acyclovir and methylprednisolone	Died
14	(25)	59	M	Swineherd	Hebei	Fever, seizures, apathy, vomiting, disturbed consciousness, res piratory failure, eye involvement (Loss of bision)	CSF NGS, PRV-specific IgG in serum	Penciclovir and foscarnet sodium	mRS:3
15	(26)	44	M	Pork vendor	Anhui	Fever, seizures, uncon-sciousness	CSF and blood NGS, PCR and Sanger sequencing, blood serological	Acyclovir, dexamethasone, sodium valproate, and sodium phenobarbital	Follow simple instructions, mRS:4
16	(27)	50	M	Pig butcher	Shandong	Fever, headache, seizures, disturbed consciousness	CSF NGS	Antiepileptic, antiviral, antibiotics, IVIG, GC	Slow responses, occasional seizures, mRS:3
17	(28)	50	F	Pork cutter	Shandong	Fever, headache, memory loss, seizures, disturbed consciousness, respiratory failure	CSF NGS	Antiepileptic, antiviral, antibiotics, IVIG, GC	Ventilator-dependent, mRS:5
18	(27)	43	M	Sick pig handler	Shandong	Fever, seizures, respiratory failure, disturbed consciousness, psychosis, extremity tremors, eye involvement (Blurry vision)	CSF NGS, vitreous humor NGS	Antiepileptic, antiviral, antibiotics, IVIG, GC	Follow simple instructions, mRS:3
19	(28)	59	M	Pork cutter	Shandong	Fever, seizures, respiratory failure, disturbed consciousness	CSF NGS	Antiepileptic, antiviral, antibiotics, IVIG, GC	Ventilator-dependent, mRS:5
20	(28)	50	M	Pork cutter	Shandong	Fever, seizures, disturbed consciousness, respiratory failure, eye involvement (blindness)	CSF NGS	Antiepileptic, antiviral, antibiotics, IVIG, GC	Slow responses, mRS:3
21	(29)	44	M	Sick pig handler	Shandong	Fever, headache, seizures, disturbed consciousness, eye involvement (visual impairment)	CSF NGS	Acyclovir, mannitol, dexamethasone and IVIG	Slow responses, visual impairment, mRS 3
22	(27)	43	M	Veterinarian	Shandong	Fever, headache, seizures, coma, alternation of consciousness, respiratory failure	CSF NGS, CSF and blood PCR, blood serological	Acyclovir	Recovery
23	(14)	43	M	Swineherd	Hebei	Fever, loss of consciousness, limb convulsions, mood abnormalities, seizures, urinary incontinence, eye involvement (intermittent blurred vision)	CSF NGS	Antivirals, acyclovir and foscarnet, olanzapine combined with lorazepam, IVIG	Psychiatric disorders, intermittent blurred vision, and an impaired cognitive function
24	(11)	56	M	Pig butcher	Shandong	Fever, lost appetite, fatigue, seizure, comatose, eye involvement (blind), limbs wriggled uncontrollably	Aqueous humor NGS, serum serological	Antivirals, foscarnet	Bilateral blindness and hand tremors, walk independently
25	(30)	54	M	Pig butcher	Hunan	Fever, impaired consciousness, cough, fuzzy consciousness, headache, fecal, and urinary incontinence, facial and limb convulsions, shortness of breath	CSF, blood, and aqueous humor NGS	Phosphonoformate, Acyclovir,Methylprednisolone Sodium Succinate, prednisone	Deep coma
26	(12)	42	M	Pork vendor	Hebei	Convulsions, headache, fever, nausea and vomiting, urinary and stool incontinence, comatose, respiratory distress, eye involvement (blurred vision)	CSF NGS	Antiviral	Walk slowly without support, eyesight visibility 15 m
27	(31)	54	M	Swineherd	Hubei	Fever, poor response, lift the right upper limb involuntarily, involuntary twitching, delirious, eye involvement (blindness)	CSF NGS	Ganciclovir, Dexamethasone and IVIG	Encephalitis was controlled, the visual impairment was difficult to reverse

M, male; F, female; CSF, cerebrospinal fluid; GC, glucocorticoids; IVIG, intravenous human immunoglobulin; mRS, modified Rankin Scale, NGS, next-genetation sequencing; PCR, polymerase chain reaction.

The clinical presentation of human PRV encephalitis is an acute severe encephalitic syndrome that typically follows a biphasic course: a prodromal influenza-like illness (fever, headache, coryza, cough), followed days later by rapid neurological deterioration with impaired consciousness, generalized tonic-clonic seizures, and meningeal signs (3). This phenotype is virtually indistinguishable from HSV-1 encephalitis, and differentiation on clinical grounds alone is often unreliable (5). Neuroimaging further reinforces this overlap: PRV encephalitis characteristically involves the medial temporal lobes (hippocampus and amygdala), insular cortex, and cingulate gyrus, a distribution nearly identical to that of HSV-1 encephalitis (9). Our patient's MRI findings, showing right-sided limbic system involvement, are fully consistent with the reported imaging pattern of PRV encephalitis.

A notable feature of our case is the visual outcome. Visual impairment is not a rare complication of PRV encephalitis. In a systematic analysis of 23 cases by Fan et al. (3), severe visual impairment occurred in 60% of patients, and 17.4% were left with permanent blindness. In the present series, the proportion was similarly high, affecting approximately 40% of patients, with multiple cases progressing to bilateral blindness. In most previously reported cases, the mechanism of visual loss has been attributed to peripheral ocular pathology, including bilateral acute retinal necrosis (10), necrotizing retinitis with retinal detachment (11), and endophthalmitis (12, 13). Our case differs in several respects: the blindness became apparent only after the patient had fully regained consciousness (day 26 from symptom onset); there was no clinical evidence of retinitis or endophthalmitis; high-dose methylprednisolone pulse therapy provided no visual improvement; and the blindness persisted with no light perception over 1-year follow-up. Collectively, these features suggest a pre-dominantly central visual pathway injury rather than a primary ocular process. To our knowledge, this pattern has been described only rarely in the literature. There are currently limited established treatment options for PRV-associated visual impairment; one of the few reported cases with partial visual recovery received aggressive antiviral therapy with ganciclovir and foscarnet (14), suggesting that early ophthalmological intervention may benefit patients with peripheral involvement. Importantly, visual symptoms can be masked by impaired consciousness, and clinicians should proactively assess visual function once the patient's mental status improves.

From a diagnostic perspective, viral encephalitis remains a major challenge: over 50% of cases have no etiology identified by conventional testing (15). Routine central nervous system infection panels do not include PRV-specific PCR, and without mNGS, PRV encephalitis would likely be missed. Wilson et al. (6) demonstrated, in a multicentre study, that CSF mNGS can detect rare pathogens missed by standard assays. In our patient, mNGS established the diagnosis approximately 13 days after symptom onset, enabling a shift from empirical broad-spectrum therapy to targeted acyclovir treatment. Notably, a study reported a case in which CSF mNGS was negative on two occasions, but intraocular fluid mNGS confirmed the diagnosis (12); this finding raises the possibility that, in highly suspicious cases with negative CSF mNGS, intraocular fluid testing or repeat lumbar puncture should be considered.

The differential diagnosis of PRV encephalitis centers on HSV-1 encephalitis, given the near-identical clinical and imaging profiles. Key differentiating features may include occupational swine exposure, negative CSF HSV PCR, and positive mNGS results for PRV. Beyond HSV-1 encephalitis, the differential diagnosis should also include varicella-zoster virus (VZV) encephalitis and Japanese encephalitis virus (JEV) infection, given overlapping clinical and imaging features. VZV encephalitis, caused by reactivation of a neurotropic alphaherpesvirus, may present with or without zoster rash and typically shows lymphocytic pleocytosis with elevated CSF protein on analysis (16, 17); diagnosis relies on CSF VZV PCR or mNGS (18). JEV, a mosquito-borne flavivirus endemic to Asia and the Western Pacific, should be considered given its epidemiological link to pigs (19). JEV classically presents with high fever, altered consciousness, and seizures, with MRI revealing bilateral thalamic and basal ganglia involvement—a highly specific imaging hallmark (20, 21). Diagnosis is established by detection of anti-JEV IgM in serum or CSF, as nucleic acid testing has low sensitivity by the time of clinical onset (19, 22). In our patient, the absence of travel to endemic areas, lack of seasonal clustering, and negative corresponding tests reasonably excluded these diagnoses. Autoimmune encephalitides (e.g., anti-NMDAR or anti-LGI1 encephalitis), tuberculous meningitis, and cryptococcal meningitis were reasonably excluded in our patient by negative corresponding tests.

Regarding treatment, it must be emphasized that no specific antiviral agent has been established or evidence-based for human PRV encephalitis to date. The current understanding of therapeutic options is derived exclusively from case reports and small case series, which limits the generalizability of any treatment recommendations. Acyclovir (typically 10–15 mg/kg every 8 h) has been the most commonly reported empirical regimen. In the present series, treatment regimens varied considerably: some patients received acyclovir alone, while others received combination therapy with foscarnet or ganciclovir, and a substantial proportion received adjunctive corticosteroids and/or IVIG. Despite these interventions, the overall outcomes remained poor. In the series of 23 cases reported by Fan et al.(3), the mortality rate was approximately 17.4 and 65.2% of survivors had severe central nervous system sequelae. In the present 27-case series, the mortality was 14.8% and the rate of severe neurological disability (mRS 4–5 or vegetative state) was 37.0%, broadly consistent with previous reports. The role of corticosteroids in viral encephalitis remains controversial; existing meta-analyses have not provided definitive recommendations (23). IVIG was used as an adjunctive immunomodulatory therapy, albeit with limited supporting evidence. In our index case, high-dose methylprednisolone pulse therapy provided no visual improvement, and the blindness persisted over 1-year follow-up. Given the absence of controlled trials, treatment decisions must be individualized, and the outcomes of this case series should be interpreted with appropriate caution regarding therapeutic efficacy. Taken together, Early diagnosis by mNGS and timely antiviral therapy appear to be the most modifiable factors for improving outcomes (1, 6).

Several limitations should be acknowledged. First, this is a single case report, and the findings may not be generalizable. Second, the exact route of PRV exposure could not be definitively traced. Third, formal ophthalmological examination, including fundoscopy and visual evoked potentials, was not performed, which limits our ability to definitively localize the visual pathway lesion. Fourth, PRV serology and serum PCR were not available at our institution, and only a single CSF mNGS test was obtained. Fifth, virus subtyping was not performed in this case. While subtyping would have provided additional epidemiological insights, the identification of PRV at the species level was sufficient for clinical diagnosis and therapeutic decision-making. Future studies with whole-genome sequencing are warranted to elucidate the genotype of PRV strains causing human infection in this region. Despite these limitations, we believe this case provides valuable clinical insights into an emerging zoonotic encephalitis with devastating visual sequelae.

## 4 Conclusion

We report a case of PRV encephalitis confirmed by CSF mNGS in a 53-year-old male, who survived but was left with permanent bilateral blindness (no light perception at 1-year follow-up). This case carries several key clinical messages: (1) PRV encephalitis should be considered in the differential diagnosis of unexplained encephalitis in patients with swine contact, and non-traditional occupational groups should not be overlooked; (2) CSF mNGS is a valuable tool for identifying PRV etiology; (3) visual impairment has been recognized as a frequent and serious complication of PRV encephalitis, and clinicians should proactively assess visual function once consciousness improves; and (4) early diagnosis and timely antiviral therapy appear to be the most modifiable factors for improving outcomes.

## Data Availability

The data that support the findings of this study are available from the corresponding author (Yonghu Zhang) upon reasonable request.

## References

[1] Wang T-Y Chao L Xue-Hui C Tongling S Yan-Dong T. Spillover of pseudorabies virus variants to humans: an urgent call for pseudorabies eradication in domestic pigs. Lancet Microb. (2026) 7:101351. doi: 10.1016/j.lanmic.2026.101351 41759550

[2] Liu Q Xiaojuan W Caihua X Shifang D Hongna Y Shibang G . A novel human acute encephalitis caused by pseudorabies virus variant strain. Clin Infectious Dis. (2021) 73 : e3690–e3700. doi: 10.1093/cid/ciaa987 32667972

[3] Fan S Hongxun Y Lei L Hongfang L Shengnan W Weili Z . Pseudorabies virus encephalitis in humans: a case series study. J. NeuroVirol. (2020) 26:556–64. doi: 10.1007/s13365-020-00855-y 32572833

[4] An T-Q Jin-Mei P Zhi-Jun T Hong-Yuan Z Na L Yi-Min L . Pseudorabies virus variant in Bartha-K61–vaccinated pigs, China, (2012). Emerg Infectious Dis. (2013) 19:1749–55. doi: 10.3201/eid1911.130177 24188614 PMC3837674

[5] Chow FC Glaser CA Heather S Xian D Messenger S Whitley R . Use of clinical and neuroimaging characteristics to distinguish temporal lobe herpes simplex encephalitis from its mimics. Clin Infectious Dis. (2015) 60:1377–1383. doi: 10.1093/cid/civ051 25637586 PMC4462661

[6] Wilson MR Sample HA Zorn KC Arevalo S Guixia Yu CLS Neuhaus J . Clinical metagenomic sequencing for diagnosis of meningitis and encephalitis. New Engl J Med. (2019) 380:2327–40. doi: 10.1056/NEJMoa1803396 31189036 PMC6764751

[7] Cao X-G Xiong-Feng Z Zha Y Chun-Yan W Min S Hua-Dong M . Diagnostic performance and clinical utility of metagenomic next-generation sequencing in suspected central nervous system infections: a prospective comparative study. Front Cell Infection Microbiol. (2025) 15:1612628. doi: 10.3389/fcimb.2025.1612628 40861498 PMC12375686

[8] Liu Q Yan K Yafei L Huihui G Chuyue Z Shibang G . The epidemiology and variation in pseudorabies virus: a continuing challenge to pigs and humans. Viruses. (2022) 14:1463. doi: 10.3390/v14071463 35891443 PMC9325097

[9] Granerod J Ambrose HE Davies WS Clewley JP Walsh AL Morgan D . Causes of encephalitis and differences in their clinical presentations in England: a multicentre, population-based prospective study. Lancet Infectious Dis. (2010) 10:835–844. doi: 10.1016/S1473-3099(10)70222-X 20952256

[10] Hu F Jiawei W Xiao-Yan P. Bilateral necrotizing retinitis following encephalitis caused by the pseudorabies virus confirmed by next-generation sequencing. Ocular Immunol Infl. (2021) 29:922–925. doi: 10.1080/09273948.2019.1704027 32735477

[11] Zhang Y Lei F Yi Z Yong Z Bing L Chuanzhu Y . A case report of long-delayed diagnosis of pseudorabies virus encephalitis with endophthalmitis: lessons from metagenomic next generation sequencing. BMC Neurol. (2023) 23:192. doi: 10.1186/s12883-023-03227-1 37194001 PMC10186779

[12] Yue L Li Y Tong F Tian M Li M Wang L . Human encephalitis complicated with ocular symptoms associated with pseudorabies virus infection: a case report. Front Neurol. (2022) 13:878007. doi: 10.3389/fneur.2022.878007 35614923 PMC9125146

[13] Ai J-W Shan-Shan W Qi C Peng C Yong-Jun L Hong-Long W . Human endophthalmitis caused by pseudorabies virus infection, China, 2017. Emerg Infect Dis. (2018) 24:1087–1090. doi: 10.3201/eid2406.171612 29774834 PMC6004832

[14] Hou Y Wang Y Zhang Y Yu H Zhao Y Yi A. Human encephalitis caused by pseudorabies virus in China: a case report and systematic review. Vector Borne Zoonotic Dis. (2022) 22:391–396. doi: 10.1089/vbz.2022.0002 35736787

[15] Venkatesan A Tunkel AR Bloch KC Lauring AS Sejvar J Bitnun A . Case definitions, diagnostic algorithms, and priorities in encephalitis: consensus statement of the international encephalitis consortium. Clin Infec Dis. (2013) 57:1114–1128. doi: 10.1093/cid/cit458 23861361 PMC3783060

[16] Tang J Kaimeng W Haoming X Jingzhe H. Metagenomic next-generation sequencing of cerebrospinal fluid: a diagnostic approach for varicella zoster virus-related encephalitis. Front Cell Infec Microbiol. (2024) 14:1509630. doi: 10.3389/fcimb.2024.1509630 39776437 PMC11703743

[17] Al-Muwallad NT Ahmed S Al-Dhahi H Aljaidi K Maram MA. Atypical presentation of varicella-zoster virus encephalitis: a case report. Cureus. (2024) 16:e68926. doi: 10.7759/cureus.68926 39381450 PMC11459875

[18] Wu H Wang R Li Y Sun X Li J Bi X. Cerebrovascular complications after adult-onset varicella-zoster virus encephalitis in the central nervous system: a literature review. Neuropsychiatr Dis Treat. (2022) 18:449–62. doi: 10.2147/NDT.S343846 35250269 PMC8890429

[19] Jindal N Bipin M Kanak R Kanak A Kumar K Pandey S . A comparative analysis of Japanese encephalitis virus (JEV) in Asia: patterns of transmission, clinical diagnosis, and control strategies. Clin Microbiol Newsletter. (2025) 53:14–19. doi: 10.1016/j.clinmicnews.2025.09.004

[20] Okada Y Toshinori O Keiichi N Nasa O Fumi K Hirotake N . Japanese encephalitis with a characteristic neuropathological distribution following early MRI-based diagnosis: an autopsy case. Neuropathology. (2026) 46:e70068. doi: 10.1111/neup.70068 42400173

[21] Keng LT Chang LY. Japanese encephalitis. CMAJ. (2018) 190:E657. doi: 10.1503/cmaj.171341 29807939 PMC5973888

[22] Ayhan N Pezzi L Do H Rego M Thy S Devatine G . Report of two severe cases of Japanese encephalitis virus in travelers returning to France, 2023-2024: clinical, diagnostic, and public health implications. ASM Case Rep. (2025) 1:e00054–e00024. doi: 10.1128/asmcr.00054-24 41244313 PMC12530225

[23] Hodzic E Rodrigo H Alejandro G Anna R Tröscher H Wagner T . Steroids for the treatment of viral encephalitis: a systematic literature review and meta-analysis. J Neurol. (2023) 270:3603–3615. doi: 10.1007/s00415-023-11715-0 37060361 PMC10105360

[24] Zhao WL Wu YH Li HF Li SY Fan SY Wu HL . Clinical experience and next-generation sequencing analysis of encephalitis caused by pseudorabies virus. Zhonghua Yi Xue Za Zhi. (2018) 98:1152–7. doi: 10.3760/cma.j.issn.0376-2491.2018.15.006 29690727

[25] Zheng L Liu X Yuan D Li R Lu J Li X . Dynamic cerebrospinal fluid analyses of severe pseudorabies encephalitis. Transbound Emerg Dis. (2019) 66:2562–2565. doi: 10.1111/tbed.13297 31325376

[26] Wang D Tao X Fei M Chen J Guo W Li P . Human encephalitis caused by pseudorabies virus infection: a case report. J Neurovirol. (2020) 26:442–448. doi: 10.1007/s13365-019-00822-2 31898060 PMC7223082

[27] Yang H Han H Wang H Cui Y Liu H Ding S. A case of human viral encephalitis caused by pseudorabies virus infection in China. Front Neurol. (2019) 10:534. doi: 10.3389/fneur.2019.00534 31214104 PMC6558170

[28] Yang X Guan H Li C Li Y Wang S Zhao X . Characteristics of human encephalitis caused by pseudorabies virus: a case series study. Int J Infect Dis. (2019) 87:92–99. doi: 10.1016/j.ijid.2019.08.007 31408708

[29] Wang Y Hong N Ziwei L Wenjuan W Xin W Yan C. Human encephalitis complicated with bilateral acute retinal necrosis associated with pseudorabies virus infection: a case report. Int J Infectious Dis. (2019) 89:51–54. doi: 10.1016/j.ijid.2019.09.019 31562933

[30] Yan W Zhiping H Yingchi Z Xiaomei W Hainan Z. Case report: metagenomic next-generation sequencing for diagnosis of human encephalitis and endophthalmitis caused by pseudorabies virus. Front Med. (2022) 8:753988. doi: 10.3389/fmed.2021.753988 35096860 PMC8795075

[31] Huang H Na W Zhi-Bing A Jun C Wei H Yi B. Cross species transmission of pseudorabies virus leads to human encephalitis and visual impairment: a case report. Front. Neurol. (2022) 13:950931. doi: 10.3389/fneur.2022.950931 36203976 PMC9530976

[32] Zhou Y Chong N Han W Yong L Meihong Z Zunchun X . Human viral encephalitis associated with SUID herpesvirus 1. Neurol Sci. (2022) 43:2681–2692. doi: 10.1007/s10072-021-05633-0 34647219 PMC8514202

